# Supplementary material for: Accessing Fungal Contributions to the Birch Effect: Real-Time Respiration from Pore-Scale Microfluidics
Source: Microorganisms. 2024 Nov 12;12(11):2295. doi: 10.3390/microorganisms12112295 (PMC11596943; doi:10.3390/microorganisms12112295)
Supplement: Supplementary file 1 [file microorganisms-12-02295-s001.zip › microorganisms-3290383-supplementary.pdf]

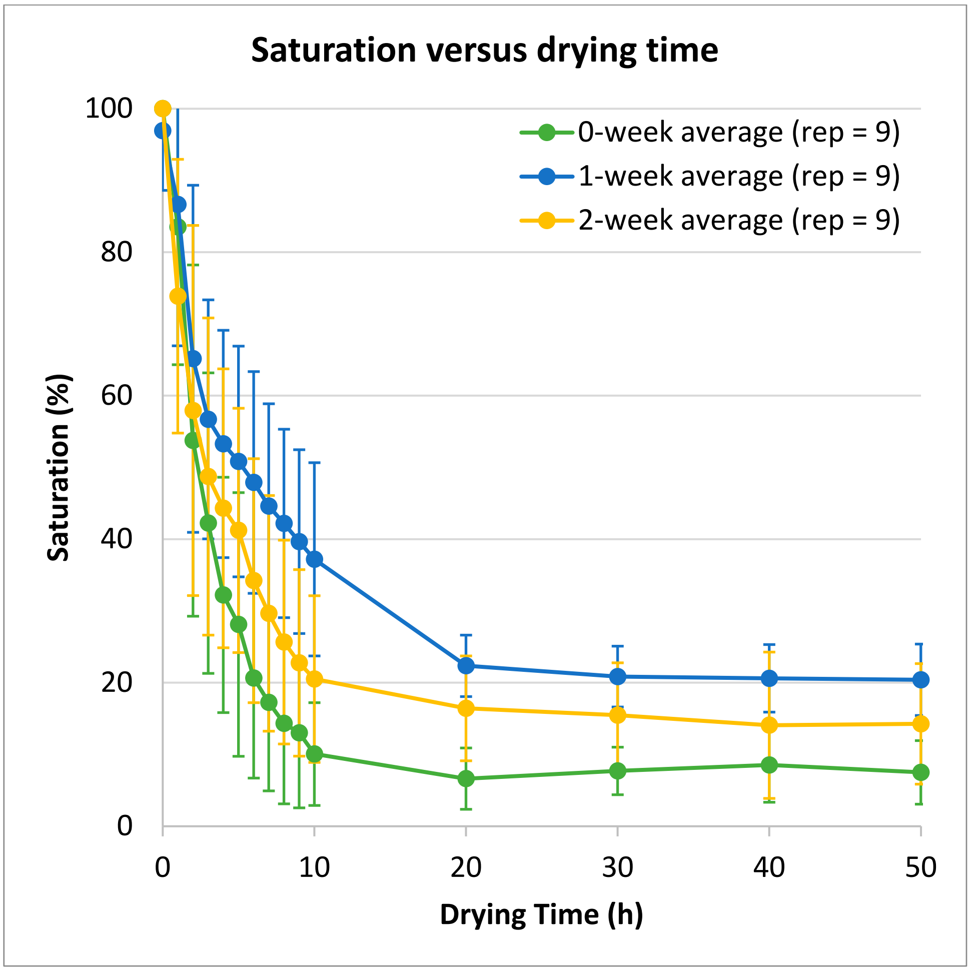

Figure S1. Monitoring the drying behavior of all fungal samples. All samples were subsequently monitored for rewetting using RTMS.
